# Detection of Tumor Recurrence via Circulating Tumor DNA Profiling in Patients with Localized Lung Cancer: Clinical Considerations and Challenges

**DOI:** 10.3390/cancers13153759

**Published:** 2021-07-26

**Authors:** Bryan Ulrich, Anne Pradines, Julien Mazières, Nicolas Guibert

**Affiliations:** 1 Internal Medicine Department, Massachusetts General Hospital, Boston, MA 02114, USA; bryulrich@gmail.com; 2 Cancer Research Centre of Toulouse (CRCT), Inserm, National Scientific Research Centre (CNRS), 31100 Toulouse, France; Pradines.Anne@iuct-oncopole.fr (A.P.); mazieres.j@chu-toulouse.fr (J.M.); 3 Medical Laboratory, Claudius Regaud Institute, Toulouse University Cancer Institute (IUCT-O), 31100 Toulouse, France; 4 Pulmonology Department, Hôpital Larrey, University Hospital of Toulouse, 31059 Toulouse, France

**Keywords:** liquid biopsy, NSCLC, non-small cell lung cancer, circulating tumor DNA, minimal residual disease

## Abstract

**Simple Summary:**

Circulating tumor DNA is a novel biomarker with emerging uses in the clinical care of patients with cancer, including non-small-cell lung cancer. Already approved for use in various clinical settings in patients with metastatic non-small-cell lung cancer, recent research has focused on the ability of circulating tumor DNA to predict relapse of patients with localized disease after treatment with curative intent. Identifying patients at increased risk of relapse after treatment with curative intent remains challenging, but several groups have identified circulating tumor DNA kinetics as a potential means of aiding our risk stratification. Herein, we discuss current research that identifies longitudinal circulating tumor DNA kinetics as a highly sensitive and specific marker for relapse. Then, we identify important clinical considerations and challenges for moving forward with further studying and eventually using this biomarker for patients with localized disease in clinic.

**Abstract:**

Approximately 30% of patients with non-small-cell lung cancer (NSCLC) present with localized/non-metastatic disease and are eligible for surgical resection or other “treatment with curative intent”. Due to the high prevalence of recurrence after treatment, adjuvant therapy is standard care for most patients. The effect of adjuvant chemotherapy is, however, modest, and new tools are needed to identify candidates for adjuvant treatments (chemotherapy, immunotherapy, or targeted therapies), especially since expanded lung cancer screening programs will increase the rate of patients detected with localized NSCLC. Circulating tumor DNA (ctDNA) has shown strong potential to detect minimal residual disease (MRD) and to guide adjuvant therapies. In this manuscript, we review the technical aspects and performances of the main ctDNA sequencing platforms (TRACERx, CAPP-seq) investigated in this purpose, and discuss the potential of this approach to guide or spare adjuvant therapies after definitive treatment of NSCLC.

## 1. Introduction

Lung cancer remains the leading cause of cancer-associated death in the United States, with over 200,000 diagnoses and 150,000 deaths from lung cancer per year [1,2]. Non-small-cell lung cancer (NSCLC) accounts for nearly 85% of these diagnoses and deaths, with small-cell lung cancer (SCLC) accounting for a majority of the remaining 15%. Approximately 30% of patients with NSCLC present with localized/non-metastatic disease for which they are eligible to undergo surgical resection or other “treatment with curative intent”, often including a localized therapy (e.g., radiation) and a course of chemotherapy [3,4,5]. Though the treatment of localized lung cancer offers opportunity for a cure, minimal residual disease (“MRD”) can occur; this is when a small amount of cancer cells remain in the body at local or distant sites, which can then grow over time [6,7]. Unfortunately, recurrence after treatment with curative intent remains a major issue and portends poor outcomes [8,9,10,11,12,13,14,15,16,17,18].

Due to the high prevalence of, and poor outcomes associated with, recurrence after treatment with curative intent, the use of adjuvant therapies is standard care for most patients with non-metastatic, stage II-IIIA NSCLC. Patients with stage IB NSCLC with high-risk features are also recommended to undergo adjuvant therapy; however, chemotherapy has been shown to worsen outcomes in patients with stage IA NSCLC after treatment with curative intent [1,19,20,21,22]. Currently, the majority of patients with stage II and IIIA NSCLC and about a quarter of patients with stage IB NSCLC receive adjuvant chemotherapy in the United States [23,24]. However, the effect of adjuvant chemotherapy is modest, with just a 5-year absolute benefit of around 5% in terms of overall death [19]. Though most adjuvant therapy is now with cisplatin-based doublet chemotherapy combinations, third-generation *EGFR* inhibitor, osimertinib, was recently shown to dramatically improve disease-free survival in patients with resectable *EGFR*-mutant NSCLC. The addition of adjuvant osimertinib (versus placebo) was associated with decreased disease recurrence or death (HR = 0.17) after a 24-month follow-up [25]. Trials investigating the use of other targeted therapies or immunotherapy continue in the adjuvant setting for patients with NSCLC continue [26,27]. With the expansion of the criteria to perform low-dose CT screening for lung cancer, the number of patients detected with resectable NSCLC will increase, thus improving adjuvant therapies which is desperately needed [28,29,30]. Investigations into technologies that identify and quantify circulating-tumor DNA, free-floating DNA in the plasma, or other bodily fluids after shedding from tumor cells have increased exponentially over the past five years [31,32,33,34,35,36,37,38,39]. The potential clinical applications range from early detection of cancer to the use of ctDNA to understand treatment response and the ultimate development of resistance [40,41,42,43,44]. Furthermore, ctDNA has been investigated in many cancer types as a biomarker to predict MRD, including in patients with colorectal or breast cancer [45,46,47,48,49,50].

Recently, two groups have published studies that make use of new sequencing and bioinformatics technologies to understand the performance of ctDNA analysis to predict MRD in patients with resectable NSCLC. In 2017, Abbosh et al. published a study that uses patient-specific multiplex PCR panels to longitudinally track tumor-associated mutations in the plasma of patients with NSCLC after treatment with curative intent [51,52,53,54]. Furthermore, in 2017, Chaudhuri et al. published a study using a next-generation sequencing panel targeting recurrently mutated genes in NSCLC to track ctDNA changes in patients with NSCLC treated with curative intent [45,55,56]. In this paper, we review these studies and then discuss future clinical considerations and challenges associated with the use of post-treatment ctDNA profiling to assess for MRD.

## 2. TRACERx

The “Tracking Non-Small-Cell Lung Cancer Evolution Through Therapy (Rx)” (TRACERx) project is a multicenter prospective study to understand the clonal evolution of NSCLC tumors from diagnosis through death (NCT01888601) [51,52,53,54]. It aims to enroll over 800 patients with stage IA-IIIA NSCLC and will track tumor genetics at various stages of the patients’ clinical courses through multiregion whole-exome sequencing. In 2017, after the enrollment and analysis of the first 100 TRACERx patients, the group published initial findings. Part of these preliminary findings was an analysis of ctDNA in patients before and at various timepoints after the surgical resection of their tumors.

**Figure 1:**
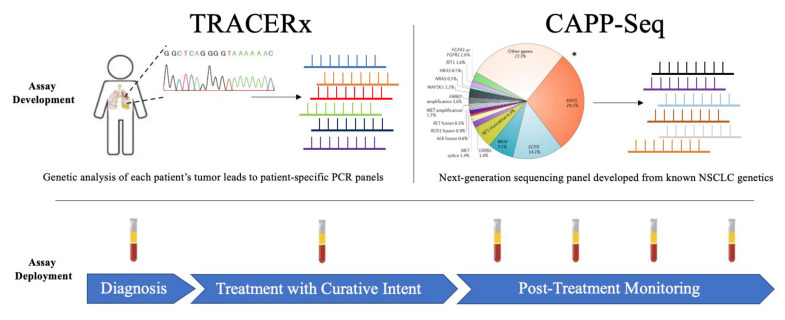
The unique assay development of TRACERx and CAPP-Seq highlight their several differences which lay the foundation for their respective strengths and weakness. However, the deployment of each assay consisted of plasma monitoring from diagnosis to the post- treatment period. * Figure adopted from Skoulidis et al. [57].

For ctDNA profiling, the group designed patient-specific multiplex-PCR panels with which they assayed plasma (Figure 1). The individualized panels were designed to target each patient’s mutational landscape, which was identified from their multiregion exome sequencing data of the primary tumor after resection. Both clonal and subclonal single nucleotide variants (SNVs) were targeted in the assay. Though the number of SNVs assayed in a given patient depended on each patient’s mutational landscape, a median of 18 patient-specific SNVs was assessed. This allowed the researchers to assess for the presence of ctDNA (defined as at least two unique cancer-associated SNVs identified in the plasma) and, if present, to quantify the variant allele frequency (VAF) of ctDNA in the plasma. The assay has high analytical sensitivity (>99% down to 0.1% variant allele frequency) and high specificity (99.6% for identification of a single SNV).

In this cohort, Abbosh et al. studied 96 of the first 100 patients, with four patients excluded due to one or more of their samples failing quality control or the multiplex PCR. This 96-patient cohort predominately included patients with stage I (61.5%) and stage II (24.0%) NSCLC, though it did include 13 patients with stage IIIA and one patient with stage IIIB NSCLC. Furthermore, 60% of these patients had adenocarcinoma, with squamous cell carcinoma accounting for 32% of tumors. A subset of 24 patients was longitudinally followed over a median of 775 days and had longitudinal ctDNA draws studied to assess for the ability of ctDNA profiling to predict post-surgical recurrence. This group included 10 patients with stage I, 10 patients with Stage II, and four patients with stage III NSCLC. Of these 24, 10 patients were relapse-free during this follow-up time, and 14 patients had confirmed relapse.

Of the 14 patients with confirmed relapse, longitudinal ctDNA analysis via patient-specific multiplex PCR predicted recurrence in 13 (93%) cases at or prior to clinical relapse was assessed via imaging. Of the 10 patients in whom clinical relapse did not occur, ctDNA profiling identified over 2 SNVs shortly after surgery, and thus was a false positive. However, clonal and subclonal ctDNA VAFs decreased during adjuvant chemo- and radiotherapy to ultimately become undetectable at around seven months post-surgery. The median lead-time between ctDNA predicting recurrence and recurrence as detected via CT scan was 70 days, though it ranged from just 10 days to nearly a year prior to imaging determined recurrence. Additionally, the researchers identified three patients, each with post-surgical ctDNA present, whose ctDNA levels increased during the period in which they received adjuvant therapy, indicating that their tumor was resistant to this therapy. Overall, recurrence of ctDNA after surgery with curative intent was found to be a highly sensitive and specific marker of clinical recurrence as assessed via imaging.

The TRACERx study should be applauded for the diversity of staging in its patient cohort, which includes a large subcohort of patients who have stage I disease. A majority of the initial 100 patient cohort were stage I patients. Due to the minimal tumor burden, this cohort presents the biggest challenge to ctDNA detection, both before surgery and if locoregional or distal recurrence occurs. Another strength of the study is the interval ctDNA analyses which occurred during adjuvant therapy treatment for some patients. This analysis led to the surprising finding that some patients had minimal residual disease resistant to adjuvant chemotherapy. This finding would be clinically useful as it may allow clinicians to change the type of adjuvant chemotherapy that their patients are receiving based off of ctDNA changes during the adjuvant therapy period.

One limitation to this study is the small number of confirmed relapses. Though ctDNA performed well at identifying patients with relapse, it is hard to make strong conclusions with just 14 total relapses and a cohort of just ten patients without relapse. However, as the TRACERx study matures, we look forward to a larger analysis of the performance of ctDNA at identifying minimal residual disease in patients with various stages of lung cancer being treated with curative intent.

## 3. CAPP-Seq

The Cancer Personalized Sequencing by Deep Sequencing (CAPP-Seq) is a ctDNA profiling platform developed by Stanford researchers to aid in the detection and quantification of tumor variants in the plasma of cancer patients. In 2017, Chaudhuri et al. published work that evaluated the ability of CAPP-seq to predict tumor recurrence in patients with non-metastatic lung cancer who have undergone treatment with curative intent [56].

To assess ctDNA, the group designed a next-generation sequencing panel covering 128 driver and passenger genes (188 kbp) known to be most commonly mutated in cancer (Figure 1) [58,59,60]. Both “driver” and “passenger” gene alterations were used in this assay, with a majority of gene mutations consisting of “passenger” mutations. After employing several advances in mutation calling of sequencing panels, their assay had remarkably strong analytic sensitivity and specificity down to a variant allele frequency of 0.02%. Additionally, their assay was previously shown to identify 50% of Stage I NSCLC, 100% of Stage II-IV NSCLC, and to have 96% specificity down to this lower limit of detection of 0.02% VAF. Of note, one major strength of this approach is that it does not require knowledge of patient-specific tumor alterations, and thus does not need to be customized.

In their work, Chaudhuri et al. apply their CAPP-Seq assay to a cohort of 40 patients with localized lung cancer that underwent treatment with curative intent. Samples were collected longitudinally, including before treatment and each two to six months thereafter, with plasma collected and analyzed at each post-treatment surveillance imaging time. Of this group, 32 patients had NSCLC, of which 21 (65%) had stage III disease, 6 had stage II disease, and 5 had stage I disease. This subset of NSCLC patients was predominately treated with both chemotherapy and radiotherapy, with just two of these patients undergoing surgical resection. In 32 of their original 40 patients, they were able to assess for the presence and quantity of ctDNA at a prespecified “MRD landmark”, which was the first blood draw collected after completion of the treatment with curative intent (up to 4 months post-treatment); this often matched up with the timing of the first post-treatment surveillance scan.

The landmark analysis showed that ctDNA was detected in plasma in 17 of the 32 patients at the first post-treatment blood draw, while 15 patients had undetectable ctDNA. Each of the 17 patients with detectable ctDNA post-treatment recurred, with over half of these patients recurring fewer than 12 months after treatment. Of the 15 with undetectable ctDNA at the MRD landmark, only one recurred with progression identified at about 8 months after the MRD landmark. As expected, patients with detectable ctDNA post-treatment had significantly worse overall survival and disease-specific survival. The ability of ctDNA detection post-treatment to predict ultimate progression was appreciated across patients with stage I-III and a type of treatment with curative intent. Additionally, Chaudhuri et al. describe a case of a patient with a targetable mutation (*EGFR* L858R) identified at MRD, which suggests that post-treatment ctDNA analysis may aid in choosing targeted therapies for patients with MRD.

This determination of genetic drivers of resistance at the point of ctDNA relapse confirming minimal residual disease is one of the biggest strengths of this study. Ultimately, when a patient experiences relapse (either as suggested via ctDNA or confirmed radiologically), genetic analysis is needed to assess for targetable mechanisms of resistance. Therefore, it is important that Chaudhuri and colleagues were able to find targetable mutations at the time of ctDNA relapse; this would allow for early changes to treatment strategy that would best target the minimal residual disease.

As discussed in the TRACERx subsection above, one weakness of this study is also the total number of relapses identified and studied. For ultimate clinical use of these technologies, far more patients and relapses needed to be studied to assess the clinical utility of ctDNA analysis in detecting minimal residual disease. Furthermore, this study primarily focused on later-stage patients, a majority being stage III with just a handful of stage I patients. In the future, more patients with stage I disease should be studied, as this is an increasingly common presentation in the era of lung cancer screening via low dose CT imaging.

The approach by Chaudhuri and colleagues of a non-personalized approach, in comparison to the personalized panels developed by the TRACERx team, has both strengths and weaknesses. The use of a standardized panel makes for a far cheaper, quicker, and resource-intensive approach because it does not require the construction of a patient-specific panel for each patient. Though it betters the TRACERx approach in cost and resource utilization, it falls behind in terms of sensitivity. The patient-specific panels developed for TRACERx patients allow for a more sensitive detection of ctDNA (when present), and thus earlier assessment of minimal residual disease.

## 4. Discussion: Future Opportunities and Challenges

**Figure 2:**
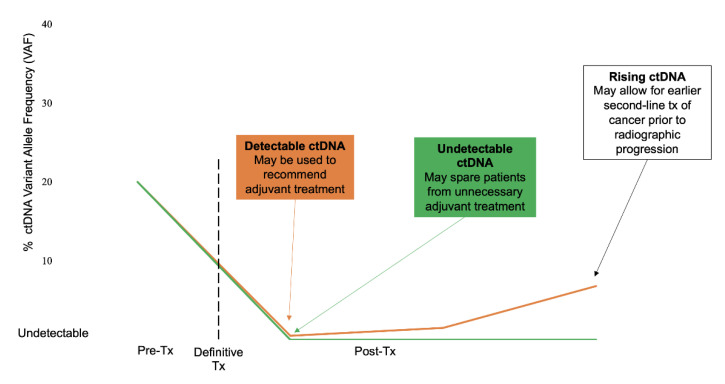
Strategies using ctDNA profiling differ between patients with detectable ctDNA indicating MRD (orange line) and undetectable ctDNA indicating treatment success (green line).

To assess the potential benefits to using ctDNA profiling to predict for MRD, it is important to consider the current clinical landscape. As detailed above, it is currently standard care to recommend adjuvant chemo- (or targeted) therapy to patients with stage IB-IIIA NSCLC treated with curative intent. Thus, the use of ctDNA profiling could have no effect on increasing the amount of adjuvant chemotherapy in these groups, as it is recommended for all of these patients. Given that the major issue with adjuvant chemotherapy is its relative lack of efficacy in the setting of high rates of recurrent disease, it is unlikely that ctDNA profiling can improve cancer-associated outcomes for many patients. Though some patients with early-stage NSCLC treated with curative intent do ultimately recur, many patients do experience cures with no cancer recurrence. It is possible that ctDNA profiling may best aid in sparing this group of patients from receiving systemic chemotherapy, which of course is associated with negative side effects. We may imagine that undetectable ctDNA at a landmark time after surgery, as detailed by Chaudhuri et al., may identify a group of patients that do not need to undergo systemic therapy. To further investigate this, prospective clinical trials are needed. Given the high rate of specificity of ctDNA profiling, such trials will hope to identify that a group of patients with undetectable ctDNA after therapy with curative intent do not benefit from systemic chemotherapy. Strategies using ctDNA profiling that differ between patients with detectable ctDNA indicating MRD and undetectable ctDNA indicating treatment success are proposed in Figure 2.

Longitudinal analysis of ctDNA may also allow for treatment intensification in patients who are refractory to adjuvant chemotherapy. Several patients in the TRACERx cohort were shown to have increasing ctDNA levels during adjuvant chemotherapy, suggesting that their cancer was refractory to this therapy. Therefore, if ctDNA profiling suggests worsening disease, as defined by increasing VAF, then both changing the treatment regimen and perhaps lengthening the course of adjuvant therapy may improve patient outcomes. Future investigations should elucidate the best clinical options to improve outcomes in patients with worsening ctDNA profiles during adjuvant therapy.

This raises the question of how to proceed clinically if imaging is stable (or improving) with worsening ctDNA analysis. Does treating at ctDNA recurrence improve survival relative to waiting for radiologic progression? We will need to define the proper clinical workup to perform in the case of worsening ctDNA profile. Imaging sites of the primary tumor after radiotherapy or surgical resection often shows post-treatment changes which conflate an analysis of local recurrence [61,62,63]. Therefore, ctDNA profiling may aid in the analysis of equivocal imaging for potential local recurrence. To assess for distant recurrence, more specialized imaging, such as PET scans, may be needed. Ultimately, in the setting of a worsening ctDNA profile, it is imperative to distinguish local from distant recurrence as the available treatment options would be different between those clinical situations. Future prospective trials should also aim to answer the most appropriate way to identify the site of recurrence.

## 5. Conclusions

Therapy after definitive treatment of early-stage NSCLC remains challenging, with suboptimal outcomes for patients. Recurrence after such therapy remains prevalent, with poor outcomes in patients who experience recurrent disease. Current adjuvant therapy options remain limited, though the recent approval of osimertinib for adjuvant therapy of early-stage EGFR-mutated NSCLC was a major success at decreasing rates of recurrence. Two recent studies identify ctDNA analysis as a strongly sensitive and specific marker which can predict likelihood of recurrence in patients who have undergone treatment with curative intent. Future use of this novel biomarker depends on prospective clinical trials investigating the clinical utility and its most appropriate place in our clinical tool arsenal.
